# The influence of gratitude on patriotism among college students: a cross-sectional and longitudinal study

**DOI:** 10.3389/fpsyg.2024.1278238

**Published:** 2024-01-26

**Authors:** Yunjun Hu, Huilin Zhang, Wei Zhang, Qian Li, Guanyu Cui

**Affiliations:** ^1^Department of Students’ Affairs, Wenzhou University of Technology, Wenzhou, China; ^2^Department of Psychology, School of Education, Wenzhou University, Wenzhou, China; ^3^Research Center for Psychology and Behavior, Wenzhou University, Wenzhou, China; ^4^Henan Industry and Trade Vocational College, Zhengzhou, China

**Keywords:** socioeconomic status, general life satisfaction, gratitude, patriotism, mediating role, moderating role

## Abstract

**Introduction:**

Patriotism, a positive emotional attachment to one’s country, has been associated with prosocial behavior, social responsibility, and gratitude. It plays a crucial role in promoting social harmony and national development. However, the factors influencing patriotism and their mechanisms remain unclear. This research consists of two studies exploring the internal mechanisms that connect gratitude and patriotism.

**Methods:**

Study 1 conducted a cross-sectional analysis among 3,826 college students to investigate the influence of gratitude on patriotism, emphasizing the mediating role of general life satisfaction and the moderating impact of socioeconomic status. This approach aimed to elucidate the complex relationships between these variables within college students. Study 2 adopted a longitudinal approach, surveying 905 college students across three-time points. This study was designed to explore the temporal mediation of general life satisfaction in the gratitude-patriotism relationship, offering insights into the evolution of these constructs over time. The sequential surveys aimed to capture the dynamic nature of gratitude’s impact on patriotism, considering the continuous interplay with general life satisfaction among college students.

**Results:**

Study 1 reveals a noteworthy finding: Gratitude enables the direct prediction of patriotism, while additionally, general life satisfaction plays a role between them. Furthermore, the predictive effect of gratitude on patriotism is strengthened among individuals with higher levels of socioeconomic status. However, there is no significant moderating effect between general life satisfaction and patriotism by socioeconomic status. Study 2 demonstrates that general life satisfaction plays a significant mediating role in the relationship between gratitude and patriotism, over a period of three times. However, the moderating influence of socioeconomic status was not substantiated in the longitudinal mediation model.

**Conclusion:**

These two studies shed light on the complex relationship between gratitude and patriotism. They emphasize the significance of gratitude, general life satisfaction, and socioeconomic status in shaping patriotism, offering potential avenues for understanding the internal mechanisms that influence patriotism.

## 1 Introduction

Patriotism refers to an emotional attachment to an in-group, signifying a sense of belonging, responsibility, and pride (Mummendey et al., 2001). It encompasses an individual’s positive feelings and attachment to their country (Osborne et al., 2017). National identity involves a citizen’s cognitive recognition of their political community and their inclination to accept the nation’s political, cultural, and ethnic values (Wodak, 2009; Huang et al., 2023). Patriotism is thought to stem from a sense of national identity (Blank and Schmidt, 2003). Therefore, an individual’s political attitudes, values, and participation also represent manifestations of patriotism. Patriotism holds considerable importance for both the nation and the individual, acting as a vital factor in the development of civic relations in a mature country (Spinner-Halev and Theiss-Morse, 2003). It also reflects an individual’s identification with and pride in their country’s shared culture, history, and political system (Ariely, 2021). Patriotism contributes to civil liberties and national security (Williams et al., 2008). Previous research has shown that patriotism plays a critical role in shaping civic attitudes and behaviors, correlating positively with various prosocial outcomes, including responses to crises (Rupar et al., 2021) and pro-environmental attitudes and behaviors (Hamada et al., 2021). Most previous studies have concentrated on the impact of patriotism on emotional and social behavior (Dražanová and Roberts, 2023), while also exploring the cultivation of patriotism in adolescents (Sharma and Hooda, 2023) and the influence of physical exercise on adolescent patriotism (Bas, 2016). However, few studies have explored the antecedent variables of patriotism. Nonetheless, evidence suggests that factors such as gratitude may influence patriotism, as gratitude can affect prosocial behavior, political attitudes, and political participation (Pang et al., 2022). These positive behaviors reflect a stronger patriotic desire (Hardin, 1993). Therefore, we hypothesize that gratitude may promote patriotism in individuals. Based on this, the present study aims to explore the intrinsic mechanism between gratitude and patriotism, thereby providing a theoretical groundwork for subsequent research endeavors.

Gratitude, originating from the Latin word gratia, signifies grace within the psychological literature (Emmons and McCullough, 2004). It has been conceptualized in various ways, such as a moral virtue, an emotional response to the kindness of others, a personality trait (Emmons et al., 2003), a broad life orientation (Wood et al., 2010), and an emotion referred to as generalized gratitude (Lambert et al., 2009). Although this study has not found direct evidence of gratitude influencing patriotism, related research still demonstrates a connection between the two. Gratitude can promote positive social behavior, which reflects an individual’s high level of patriotism (Hardin, 1993; McCullough et al., 2001). Based on moral affect theory, gratitude serves a specific moral function in driving individuals toward prosocial behavior (McCullough et al., 2001). Consequently, gratitude is associated with various essential social and individual outcomes, potentially impacting a range of positive behaviors (Lee, 2022). These encompass prosocial behavior (Bartlett and DeSteno, 2006; Grant and Gino, 2010; Pang et al., 2022; Li et al., 2023) and social justice (Yost-Dubrow and Dunham, 2018). Building on this foundation, Hardin posits that voluntary contributions for collective benefit also represent a form of altruism (Hardin, 1993). Local patriotism, which reflects a sense of belonging and attachment to one’s city or community, demonstrates altruism within the community (Hardin, 1993). Local patriots are more inclined to engage in prosocial behavior, indicating that gratitude not only promotes an individual’s prosocial behavior but also contributes to elevating their level of patriotism (Hardin, 1993). This indirect evidence suggests a correlation between gratitude and patriotism. Apart from their direct relationship, is there an intrinsic mechanism between them? Therefore, we further explore the roles of life satisfaction and socioeconomic status in the relationship between gratitude and patriotism.

Scholars have extensively investigated life satisfaction at the individual level (Easterlin, 1995; Blanchflower and Oswald, 2000), providing evidence to support the notion that life satisfaction may be a pivotal factor in the relationship between gratitude and patriotism. On one hand, a connection exists between gratitude and life satisfaction, the broaden-and-build theory of positive emotions posits that positive emotions can initiate a cascading effect, expanding individuals’ range of other positive emotions and behaviors (Fredrickson, 2001). Grounded in this theory, researchers have conducted numerous studies on gratitude and life satisfaction, discovering that gratitude positively affects individuals’ psychological wellbeing and life satisfaction (Perez et al., 2021; Lee, 2022). On the other hand, an indirect relationship exists between life satisfaction and patriotism through related variables. Individuals’ wellbeing can impact their political engagement, such as their intention to vote (Weitz-Shapiro and Matthew, 2011; Flavin and Keane, 2012). Furthermore, studies have revealed that governments frequently prioritize enhancing citizens’ wellbeing to foster active political participation (Ward, 2020; Ward et al., 2021). Nonetheless, despite the wealth of research illustrating the influence of life satisfaction on the political domain, a direct association between life satisfaction and patriotism remains to be established.

This knowledge gap highlights the need for further investigation to better understand the complex relationship between these variables. Interestingly, recent studies have revealed a complex relationship between socioeconomic status and patriotism. On one hand, Yost-Dubrow and Dunham (2018) found that individuals with lower socioeconomic status tend to report higher levels of patriotism. On the other hand, individuals with higher socioeconomic status are more likely to base their political beliefs on moral considerations (Brown et al., 2021). These findings suggest that social and economic status may play a crucial role in the link between gratitude, life satisfaction, and patriotism (Rubin and Stuart, 2018). By examining these variables as moderators, this study seeks to shed new light on the interplay between psychological wellbeing, socioeconomic status, and patriotism. Furthermore, this study will employ mediation and moderation analyses to explore the complex mechanisms underlying the relationship between gratitude, life satisfaction, and patriotism.

The primary objective of this research is to elucidate the underlying mechanisms that connect gratitude and patriotism. By examining the roles of general life satisfaction and socioeconomic status, this study aims to deepen the understanding of how gratitude influences patriotism. Specifically, the research consists of two distinct studies: The first study focuses on investigating the impact of gratitude on patriotism, taking into account the mediating role of general life satisfaction and the moderating effect of socioeconomic status. This aims to unravel the complex interplay between gratitude, life satisfaction, and patriotism, providing insights into how these variables interact to shape patriotism. Building upon the insights from the first study, the second study delves into the longitudinal dynamics of these relationships. It explores how the mediating role of life satisfaction in the gratitude-patriotism nexus evolves over three different time points, offering a comprehensive understanding of the temporal aspects of this relationship.

### 1.1 The relationship between gratitude and patriotism

Gratitude, both as a dispositional trait and a transient state of mind, has been shown to have a profound impact on individuals’ emotional wellbeing and generate a multitude of positive social outcomes (Wood et al., 2010; Bartlett et al., 2012). Additionally, this emotion reinforces individuals’ moral responsibility to assist those in need, as demonstrated by studies in the field (Wood et al., 2010; Bartlett et al., 2012). Furthermore, moral affect theory posits that gratitude serves as a specific moral function that motivates individuals to engage in prosocial behavior, further enhancing the positive impact of this emotion (McCullough et al., 2001). Recent research has suggested that individuals who exhibit more commendable social behaviors, such as prosocial and altruistic actions, tend to be more patriotic (Hardin, 1993; Rupar et al., 2021). Notably, gratitude has also been found to have a close link with national sentiment (Karakaya, 2022), providing further evidence for the potential influence of gratitude on patriotism. Therefore, gratitude may serve as a critical factor in shaping individuals’ attachment to their nation and inspire them to engage in behaviors that benefit their community and country.

Gratitude is a crucial element of a broader life orientation that involves recognizing and appreciating the positive aspects of the world (Wood et al., 2010). Grateful individuals tend to openly express their appreciation more often (McCullough et al., 2002), and this expansion of gratitude has enduring and adaptive benefits that enhance personal resources, including physical, intellectual, social, and psychological resources. Therefore, grateful individuals tend to have a more positive outlook, which may contribute to a stronger sense of national identity (Blank and Schmidt, 2003).

Although there is no direct evidence that establishes a causal relationship between gratitude and patriotism, research suggests that gratitude has positive effects on prosocial behavior, which in turn can indirectly support the hypothesis. This is because prosocial behavior, such as altruism and kindness, is associated with greater patriotism (Hardin, 1993; Rupar et al., 2021). Therefore, it is plausible to suggest that gratitude, which has been shown to promote prosocial behavior (McCullough et al., 2001), may also play a role in fostering a stronger sense of national identity and patriotism. However, further research is needed to explore this relationship in greater detail. Individuals with high levels of gratitude are likely to recognize the value of benefits and creatively express gratitude through various prosocial behaviors (Fredrickson, 2001; Li et al., 2023). Moreover, gratitude fosters various forms of relationship behavior, including social affiliation (Bartlett et al., 2012), social inclusion (Bartlett et al., 2012), perspective-taking (Gordon and Chen, 2013), and social support (Lau and Cheng, 2017). Gratitude also reduces antisocial behavior (Stieger et al., 2019). According to the moral affect theory of gratitude, beneficiaries, and donors exhibit prosocial behavior when they experience gratitude (McCullough et al., 2001; Pang et al., 2022). Thus, those who display high levels of gratitude are more likely to possess positive values and a stronger sense of national identity.

### 1.2 Gratitude, general life satisfaction, and patriotism

General Satisfaction with Life (GSL) is a crucial component of subjective wellbeing, representing the cognitive evaluation of one’s quality of life based on individual criteria (Emerson et al., 2017). When circumstances are favorable, individuals are likely to experience a high degree of life satisfaction. However, other factors, such as gratitude and patriotism, may also play a role in shaping this evaluation. Gratitude has been conceptualized in various ways and has been linked to numerous social and individual benefits, including better physical and mental health (Lavelock et al., 2016) and overall wellbeing (Wood et al., 2010; Zhang et al., 2022). The outcomes of trait gratitude include domain-specific satisfaction, and individuals with high gratitude traits tend to appreciate what they have, known as a “have focus,” leading to increased satisfaction across all areas of life (Fagley and Adler, 2012). Research has consistently shown that gratitude traits are positively associated with life satisfaction (Buschor et al., 2013), with both self-reported and peer-reported traits of gratitude linked to life satisfaction (Perez et al., 2021). Moreover, Zhang found a strong association between grateful traits and life satisfaction after a time lag of 4 weeks in one of three studies. Gratitude traits are also associated with satisfaction in other areas of life (Zhang, 2020), such as job satisfaction (Kim et al., 2019; Moon and Jung, 2020) and school satisfaction (Sun et al., 2014). On the other hand, patriotism, the love and devotion to one’s country, has also been found to correlate positively with life satisfaction (Zhang and Zuo, 2012; Gomez Berrocal et al., 2020). Thus, life satisfaction may serve as a mediator between gratitude and patriotism.

Life satisfaction has important political implications because it is associated with regime support (Chen and Shi, 2001; Stutzer and Bruno, 2006; Tang, 2016) or increasing the possibility of political participation in various types of regimes (Weitz-Shapiro and Matthew, 2011). The key to individual life satisfaction is individual pride (Chen et al., 2015) and a sense of belonging. According to social identity theory (Tajfel and Turner, 1986), an individual can identify himself as a member of a group and incorporate the meaning and value given to him by the group into his self-representation. In addition, according to the Intergroup Emotions Theory (Mackie et al., 2000), when individuals have a high sense of belonging to their collective, they will have strong emotional attachment and value attachment. Extending emotions from the individual level to the group level (Smith and Kim, 2006). When we all belong to a large collective (country), the sense of belonging of the individual becomes the sense of belonging to the country, and the sense of pride becomes the pride of the country (Zhang et al., 2020). There is a significant positive correlation between national identity and both national pride and in-group preference (Zhang and Zuo, 2012). In addition, an individual’s political values are closely related to their life satisfaction (He et al., 2022). This suggests that there is an indirect positive correlation between life satisfaction and patriotism. Therefore, it can be inferred that individuals who have a strong sense of national identity and pride, as well as those who hold values that align with their country’s ideals, are likely to experience greater life satisfaction (Liu et al., 2021). Additionally, this may have implications for policymakers and educators who wish to promote patriotism and national identity as a means of enhancing overall wellbeing and social cohesion. Thus, general life satisfaction may play a mediating role between gratitude and patriotism.

### 1.3 The moderating influence of socioeconomic status

Socioeconomic status (SES) is considered the primary determinant of class identity (Jackman and Jackman, 1973). It comprises both objective material resources or capital and the subjective experience of these resources, typically assessed through indicators such as wealth, education, and occupational prestige (Kraus et al., 2011). Moreover, a powerful socioeconomic status is also associated with a solid political and moral foundation (Brown et al., 2021). The measurement of subjective socioeconomic status relies on individuals’ perceptions of their socioeconomic status and their sense of place in the social hierarchy relative to others (Singh-Manoux et al., 2005). A person’s socioeconomic status reflects their level of access to material and social benefits from society and may also constitute a dominant or subordinate structure (Ishio, 2010).

For young people, SES mainly refers to the perceived status of their family in the social and economic class (Goodman et al., 2015). It mainly studied the determinants of class identity and the relationship between class identity, political attitudes, and behaviors (Adler et al., 2000; Pankaew et al., 2022). Patriotism is often seen as a political attitude (Martynov et al., 2020), which is people’s self-identification as citizens of a country (Martynov et al., 2020). So social class is associated with patriotism. Most previous studies have shown that socioeconomic status is negatively correlated with patriotism, and people with lower socioeconomic status will engage in more pro-social behaviors (Yost-Dubrow and Dunham, 2018) and be more patriotic (Hardin, 1993). Osborne suggests that social dominance tendencies (SDO) are closely related to patriotism (Osborne et al., 2017). He postulated that the relationship between SDO and patriotism should be positive in countries that support group-based hierarchies and dominate worldwide politics, and negative in countries that formally oppose social hierarchies and have (relatively) no global influence. The white respondents tended to belong to lower groups (Peña and Sidanius, 2002), the higher their level of American patriotism. The class in different statuses has different value orientations, behavior modes, and group living habits, resulting in the difference in value culture and benefits orientation. Socioeconomic status affects individuals’ political views and political trust and then affects individuals’ identification with the dominant values of society (Schoon et al., 2010). According to the rational Voter Model (Meltzer and Richard, 1981), a famous theory of the relationship between socioeconomic status and economic policy preferences, it is proposed that if the current national tax and welfare system is unfavorable to people, they may form negative political attitudes and reduce patriotism (Martynov et al., 2020).

Gratitude has been found to have a strong positive influence on prosocial behavior among individuals with low socioeconomic status, suggesting that both gratitude and general life satisfaction are likely to be significant factors affecting patriotism (Yost-Dubrow and Dunham, 2018). However, the influence of gratitude on individuals with high socioeconomic status requires careful consideration. While gratitude traits have been positively associated with charitable giving, an indicator of prosocial behavior, this relationship’s dependency on socioeconomic status warrants further examination (Carvalho et al., 2021). Specifically, individuals of higher socioeconomic status with pronounced gratitude traits may engage in more prosocial behaviors, yet the direct link to patriotism remains to be explored (Hardin, 1993).

Importantly, socioeconomic status has been identified as a significant predictor of an individual’s wellbeing and life satisfaction (Vayness et al., 2020), factors that are intricately linked with national identity, pride, and patriotism (Chen et al., 2015; Wei et al., 2022). It posits that individuals with lower socioeconomic status might find their sense of patriotism more influenced by their levels of gratitude and life satisfaction. In contrast, those with higher socioeconomic status might display different patterns, possibly correlating their sense of patriotism more closely with broader aspects of wellbeing and potentially gratitude traits (Chen et al., 2015).

### 1.4 The present study

**FIGURE 1:**
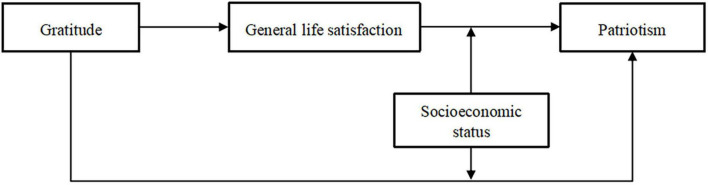
The proposed moderated mediation model.

This study draws upon the theoretical frameworks of moral affect theory (McCullough et al., 2001), Broaden-and-Build Theory (Fredrickson, 2001), and social identity theory to investigate the relationship between gratitude and patriotism through two separate research endeavors (Tajfel and Turner, 1986). Study 1 focuses on examining the impact of gratitude on patriotism while considering the mediating role of general life satisfaction and the moderating effect of socioeconomic status. By analyzing these intricate relationships, the study aims to uncover the complex interplay among gratitude, general life satisfaction, and patriotism, providing valuable insights into their interdependent dynamics. To further investigate causal relationships among several variables, building on the findings of Study 1, Study 2 takes a longitudinal approach to delve deeper into the mediating role of general life satisfaction in the association between gratitude and patriotism. This investigation explores how the effects of gratitude on patriotism evolve, offering valuable insights into the temporal dynamics of these psychological constructs. This research aims to develop a more nuanced understanding of the factors influencing patriotism by integrating various theoretical perspectives and examining the potential role of gratitude in shaping patriotism. Our study proposes a structural equation model (refer to Figure 1) to explore the hypothesized relationships. We suggest that general life satisfaction may act as a mediator in the relationship between socioeconomic status and patriotism. Additionally, we hypothesize that socioeconomic status could have a moderating effect within this mediation framework. This approach seeks to uncover the intricate dynamics between gratitude, general life satisfaction, and patriotism, contributing to the broader discourse on patriotism. Through this model, we intend to learn more about how gratitude and patriotism interact. We proposed the following three hypotheses:

H1: Gratitude has a positive predictive effect on patriotism.

H2: General life satisfaction mediates the relationship between gratitude and patriotism.

H3a: Socioeconomic status moderates the relationship between gratitude and patriotism, such that the relationship is stronger among individuals with high socioeconomic status compared to those with low socioeconomic status.

H3b: Socioeconomic status moderates the mediating effect of general life satisfaction between gratitude and patriotism, with the mediating effect being more significant in individuals with low socioeconomic status compared to those with high socioeconomic status.

## 2 Materials and methods

### 2.1 Participants and procedures

This study employed a cluster sampling method to select and analyze the sample. Participants were chosen from five colleges and universities in Henan and Zhejiang provinces. A total of 3,900 questionnaires were collected, and after excluding invalid responses, 3,826 questionnaires were collected. Among the participants, there were 1,367 male students and 2,459 female students. The average age of the participants was 19.06 ± 1.03 years, with the majority falling into the age groups of 18, 19, or 20 years, comprising 28.2%, 39.3%, and 20.4% of the sample, respectively. The study was conducted in collaboration with the Relevant education department in 2022. Data collection from all participating schools was completed within 2 weeks. Participation in the study was entirely voluntary and anonymous for both schools and students. All sampled schools willingly agreed to participate in the research. To ensure confidentiality, a dedicated period was allocated for students to complete the questionnaires. Participants were assured that their responses would be promptly sealed in envelopes to maintain confidentiality. Out of the 4,290 questionnaires distributed, 3,900 responses were collected, resulting in an impressive 91% response rate. After carefully examining the survey data, 64 responses were excluded due to patent responses or excessive missing values. As a result, a total of 3,826 valid responses were included in the final analysis. During the dissemination of research results to local stakeholders, stringent procedures were implemented to protect the confidentiality of the respondents. No information related to the participants’ affiliation was recorded to ensure their anonymity. Initially, the study design included a nested structure, with individuals nested within schools. However, to minimize the risk of respondents being identifiable to local stakeholders familiar with their schools, only the respondents’ school levels were collected. These measures were implemented to uphold ethical standards and safeguard the privacy of the study participants while ensuring the reliability and validity of the research.

In Study 2, the longitudinal sample was derived from the initial participant pool of Study 1. We specifically targeted a subset of college students who had participated in the first study. This approach was chosen to ensure continuity and relevance in the data collected over time. To select participants for the longitudinal study, we employed a stratified sampling method. This involved identifying a representative subset of students from the larger group surveyed in Study 1. The criteria for selection included a willingness to participate in multiple surveys over a year and the ability to provide consistent, reliable responses across different time points. The longitudinal study consisted of three surveys, conducted at fixed intervals. This staggered approach was designed to capture the evolving nature of the relationships between gratitude, patriotism, and general life satisfaction over time. The participants were contacted via the contact information they had provided in the initial survey, and they were briefed about the nature and purpose of the follow-up studies. To ensure a high response rate and consistent participation, we maintained regular communication with the participants and provided reminders before each survey. This careful selection and follow-up process allowed us to gather longitudinal data from a committed and reliable subset of the original sample, ensuring the validity and robustness of our findings. Among the participants, there were 240 male students and 665 female students, representing a diverse gender distribution. The age range of all participants was between 16 and 23 years old, with an average age of 18.88 ± 1.00 years. Notably, the highest proportion of participants, 37.1%, fell into the 19-year-old age group, highlighting the significance of this age cohort in the study.

### 2.2 Measurements

#### 2.2.1 Gratitude

The gratitude questionnaire comprises six items, including statements such as “There are many things in my life for which I feel grateful.” Responses are scored on a 6-point scale, ranging from “strongly disagree” to “strongly agree,” to assess individual variations in the tendency toward gratitude (McCullough et al., 2002). The higher the total scores of the questionnaire, the higher the individual’s gratitude tendency. In the cross-sectional and longitudinal samples of this study, the Clone Bach coefficient of the questionnaire ranged from 0.73 to 0.76.

#### 2.2.2 Socioeconomic status

In examining the impact of socioeconomic status on various psychological constructs, researchers frequently employ the ‘ladder scale,’ a methodological tool widely recognized in both domestic and international studies (Adler et al., 2000). This scale is particularly illustrative in the context of China, where it represents different positions of families within the societal structure. The “ladder” metaphorically depicts these positions: the higher a family is placed on the ladder, the better their overall circumstances, encompassing aspects such as financial stability, social standing, and access to resources. A 10-level score was adopted, and the subjects were asked to subjectively assess their position on the ladder where their family stands. Those who stand at the top of the ladder have the most money, the most education, and the best jobs, compared with those at the bottom.

#### 2.2.3 General life satisfaction

General life satisfaction, a key variable in studies exploring the interplay between socioeconomic status and psychological constructs, is typically measured using the General Life Satisfaction Scale. This scale comprises six items, each rated on a scale ranging from “strongly disagree” to “strongly agree.” This six-point scoring system is designed to assess an individual’s overall satisfaction with their life. A representative item from the scale, for instance, is “I am satisfied with my life.” Such statements are intended to gauge a respondent’s general sense of contentment and wellbeing. The cumulative score from these items provides a comprehensive measure of an individual’s life satisfaction, offering valuable insights into their subjective wellbeing and its potential correlations with other factors like gratitude and patriotism. The Clone Bach coefficient of the questionnaire ranged from 0.75 to 0.80 in the cross-sectional and longitudinal samples of this study (Leung and Leung, 1992).

#### 2.2.4 Patriotism

The questionnaire on patriotism in The Moral Emotion Questionnaire for College Students was 5 items in total (Huang et al., 2014). An example of an item from this section is “I believe that national interests should always be the top priority,” reflecting the depth of patriotism. Respondents rate each item on a scale that ranges from “completely inconsistent” to “completely consistent.” This scale is quantified using a six-point grading system, allowing for a nuanced assessment of the degree of patriotism. The Clone Bach coefficient of the questionnaire ranged from 0.78 to 0.92 in the cross-sectional and longitudinal samples of this study.

### 2.3 Data analysis

In this study, we used SPSS 25.0 and Mplus 8.3 to conduct data analysis. Firstly, we performed Pearson correlation analysis on gratitude, general life satisfaction, patriotism, and socioeconomic status using SPSS 25.0. Next, we used the ML estimator in Mplus 8.3 to estimate the parameters of the Structural Equation Model (SEM) and perform the mediation analysis. To test the mediating and moderating effects, we conducted repeat sampling using BC Bootstrap and estimated the 95% confidence intervals through 1,000 samples.

In this study, we employed a questionnaire survey approach, which inherently carries the risk of common method bias. To address this concern, we analyzed both cross-sectional and longitudinal data using Harman’s single-factor test. The analysis identified four factors with eigenvalues exceeding 1. The primary factor explained 31.20 and 24.75% of the variance in the cross-sectional and longitudinal datasets, respectively, which is below the 40% threshold commonly used to indicate significant common method bias. Consequently, our results suggest that common method bias is not a substantial concern in this study. Furthermore, we conducted a multicollinearity assessment. The results indicated that all Variance Inflation Factor (VIF) values were below 2, signifying that multicollinearity does not pose a significant issue in our analysis.

### 2.4 Results

#### 2.4.1 A model fit analysis

Regarding model fit, both the cross-sectional and longitudinal models demonstrated a good fit with the data. The fit indices for the cross-sectional model were: χ^2^/df = 4.72, *p* = 0.000, Comparative Fit Index (CFI) = 0.99, Tucker-Lewis Index (TLI) = 0.98, Root Mean Square Error of Approximation (RMSEA) [90% CI] = 0.03 [0.01, 0.05], and Standardized Root Mean Square Residual (SRMR) = 0.01. For the longitudinal model, the indices were: χ^2^/df = 2.57, *p* = 0.00, CFI = 0.99, TLI = 0.98, RMSEA [90% CI] = 0.04 [0.02, 0.06], and SRMR = 0.03. These results indicate robust model fits in both instances.

#### 2.4.2 Descriptive statistics

The mean, standard deviation, and correlation coefficient of each variable are as follows:

Table 1 suggests that socioeconomic status is significantly negatively correlated with patriotism. Patriotism, socioeconomic status, and gratitude are significantly positively correlated with general life satisfaction, respectively, while gratitude and general life satisfaction are significantly positively correlated with patriotism, respectively.

**TABLE 1:** Descriptive statistic of cross-sectional data (*N* = 3,826).

	M ± SD	1	2	3	4
1. Gratitude	4.38 ± 1.83	–			
2. General Life Satisfaction	4.88 ± 0.78	0.42***	–		
3. Patriotism	4.31 ± 0.93	0.51***	0.30***	–	
4. Socioeconomic Status	5.35 ± 0.74	0.09***	0.18***	−0.04*	–

*p < 0.05

***p < 0.001.

Table 2 provides a comprehensive overview of the average levels and variability of gratitude, general life satisfaction, and patriotism at three distinct time points in the study. By examining the means and variances of these variables across time, we can gain valuable insights into their temporal dynamics and how they may change over the course of the study.

**TABLE 2:** Participants’ scores for various variables in the three measurements (*N* = 905).

Variables	T1	T2	T3
Patriotism (M ± SD)	5.33 ± 0.77	5.39 ± 0.85	5.26 ± 0.87
General life satisfaction (M ± SD)	4.25 ± 0.87	4.39 ± 0.84	4.24 ± 0.81
Gratitude (M ± SD)	4.83 ± 0.78	4.58 ± 0.8	4.6 ± 0.83

Table 3 lists the correlation coefficients between the variables at the time points of the three measurements. Patriotism at each time point was significantly correlated with general life satisfaction and gratitude. This provided a basis for further analysis of the causation and mediation effects of these variables.

**TABLE 3:** Correlation analysis of variables across three-time points (T1 to T3, *N* = 905).

	1	2	3	4	5	6	7	8	9
Patriotism (T1)	1								
Patriotism (T2)	0.36***	1							
Patriotism (T3)	0.34***	0.50***	1						
General life satisfaction (T1)	0.29***	0.12***	0.20***	1					
General life satisfaction (T2)	0.22***	0.37***	0.30***	0.42***	1				
General life satisfaction (T3)	0.19***	0.23***	0.36***	0.40***	0.45***	1			
Gratitude (T1)	0.50***	0.29***	0.36***	0.41***	0.31***	0.28***	1		
Gratitude (T2)	0.32***	0.52***	0.41***	0.23***	0.36***	0.27***	0.46***	1	
Gratitude (T3)	0.29***	0.37***	0.64***	0.29***	0.33***	0.42***	0.49***	0.53***	1

***p < 0.001.

#### 2.4.3 A moderated mediation model of the relationship between gratitude and patriotism: evidence from cross-sectional samples

**FIGURE 2:**
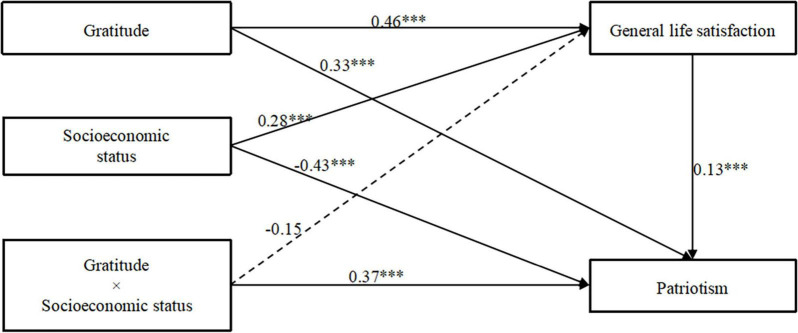
The result of the moderated mediation model. *N* = 3,826; ***P < 0.001.

The results show that the moderated mediation model is significant (*R*^2^ = 0.28, *F* = 293.57, *p* < 0.001). Gratitude significantly positively predicted patriotism (β = 0.33, *p* < 0.001, 95% CI [0.26, 0.41]), which verifies Hypothesis 1 (Figure 2). Gratitude significantly positively predicts general life satisfaction (β = 0.46, *p* < 0.001, 95% CI [0.46, 0.53]), general life satisfaction significantly positively predicts patriotism (β = 0.13, *p* < 0.001, 95% CI [0.41, 0.47]). Since the confidence interval does not contain 0, it indicates that general life satisfaction plays a partial mediating role between gratitude and patriotism, and hypothesis 2 is supported.

We found a statistically significant moderating effect of socioeconomic status on gratitude and patriotism (β = 0.37, *p* < 0.001, 95% CI [0.01, 0.03]). However, the moderating effect between general life satisfaction and patriotism was not significant. This partially supports hypothesis 3, which posited that socioeconomic status moderates the relationship between gratitude and patriotism (Table 4).

**TABLE 4:** Test of moderated mediating effects.

Variables	*B*	SE		*t*	95% CI	(Bias-corrected)
					**LLCI**	**ULCI**
Gratitude	0.33	0.04		30.31***	0.26	0.41
General Life Satisfaction (GLS)	0.13	0.02		8.48***	0.10	0.16
Socioeconomic Status (SS)	−0.43	0.10		−7.39***	−0.63	−0.23
Gratitude × SS	0.37	0.37		2.66**	0.16	0.58
*R* ^2^	0.28					
*F*	293.57					
	**Estimate**		**S.E.**		**BootLLCI**	**BootLLCI**
Direct effect Gratitude → Patriotism	0.33***		0.04		0.20	0.33
Indirect effect Gratitude → General life satisfaction→ Patriotism	0.06***		0.01		0.03	0.06
Total	0.39***		0.04		0.25	0.37

**p < 0.01

***p < 0.001.

**FIGURE 3:**
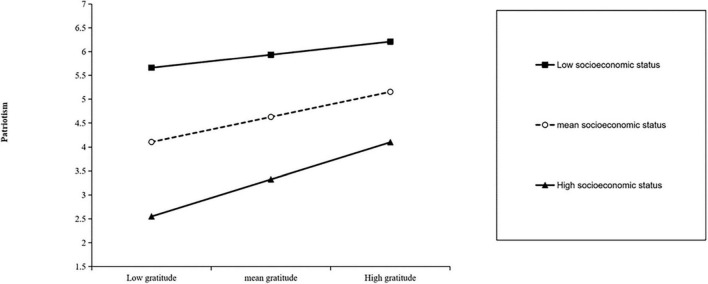
The moderating effect of socioeconomic status between gratitude and patriotism.

To understand the moderating effect of socioeconomic status on gratitude and patriotism in a more specific way, a simple slope test is needed, that is, the effect value of gratitude on patriotism is calculated according to the mean of socioeconomic status score plus or minus one standard deviation, and a simple effect analysis chart is drawn (Figure 3). According to the simple slope test, gratitude has a significant positive effect on patriotism when socioeconomic status is low (M-1SD), but its prediction effect is small (β_*simple*_ = 0.28, *P* < 0.001). When the socioeconomic status is high (M + 1SD), gratitude can also significantly positively predict patriotism (β_*simple*_= 0.78, *P* < 0.001), which indicates the socioeconomic status rises, both gratitude and patriotism rise as well.

**FIGURE 4:**
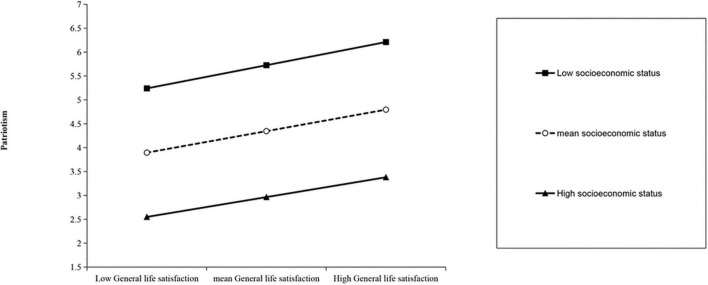
The moderating effect of socioeconomic status between general life satisfaction and patriotism.

In addition, we computed the effect size of general life satisfaction on patriotism by using the mean of the socioeconomic status score plus or minus one standard deviation. We then created a simple effect analysis chart to examine the moderating effect of socioeconomic status on the relationship between general life satisfaction and patriotism (see Figure 4). However, the results of the simple slope test revealed that general life satisfaction did not have a significant impact on patriotism when socioeconomic status was low (M-1SD) (β_*simple*_= 0.49, *P* > 0.05). Similarly, when socioeconomic status was high (M+1SD), general life satisfaction did not significantly predict patriotism (β_*simple*_ = 0.42, *P* > 0.05). This means that although the level of patriotism for both groups slightly increased with the improvement in general life satisfaction, there is no significant difference between the two groups in the level of increase.

#### 2.4.4 Cross-lagged analysis of gratitude and patriotism at three time points

**FIGURE 5:**
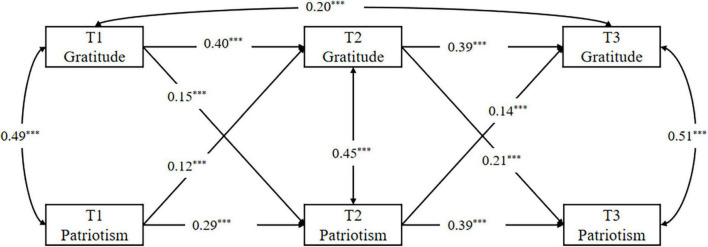
The cross-lagged model of gratitude and patriotism at T1, T2, T3. ***p < 0.001.

To further elucidate the potential causal dynamics between the independent and dependent variables, this study has developed a cross-lagged model featuring gratitude (independent variable) and patriotism (dependent variable) across three distinct time points: T1, T2, and T3. The model demonstrates robust fit indices: χ2 = 84.11, df = 13, CFI = 0.96, TLI = 0.93, RMSEA (90% CI) = 0.07 [0.06, 0.09], SRMR = 0.06. Detailed results from the cross-lagged analysis of gratitude and patriotism are presented in Figure 5. Examining the longitudinal trajectory of each variable, we observe that T1 gratitude significantly predicts T2 gratitude (β = 0.40, *p* < 0.001), and this predictive relationship continues from T2 gratitude to T3 gratitude (β = 0.39, *p* < 0.001). Similarly, T1 patriotism significantly forecasts T2 patriotism (β = 0.29, *p* < 0.001), T2 patriotism significantly forecasts T3 patriotism (β = 0.39, *p* < 0.001). When exploring the interplay between different variables over time, we find that T1 gratitude significantly forecasts T2 patriotism (β = 0.15, *p* < 0.001), and T2 gratitude continues this trend by significantly predicting T3 patriotism (β = 0.21, *p* < 0.001). Conversely, T1 patriotism significantly predicts T2 gratitude (β = 0.12, *p* < 0.01), with T2 patriotism also significantly influencing T3 gratitude (β = 0.14, *p* < 0.01).

From these findings, it is evident that gratitude and patriotism are interrelated and potentially causally linked variables. However, the primary focus of this study is to explore the impact of gratitude on patriotism and to understand the underlying mechanisms. Therefore, the following analysis and discussion will focus on the discussion of gratitude’s prediction of patriotism and its intermediary mechanism.

#### 2.4.5 The mediating effects of general life satisfaction: evidence from longitudinal samples

**FIGURE 6:**
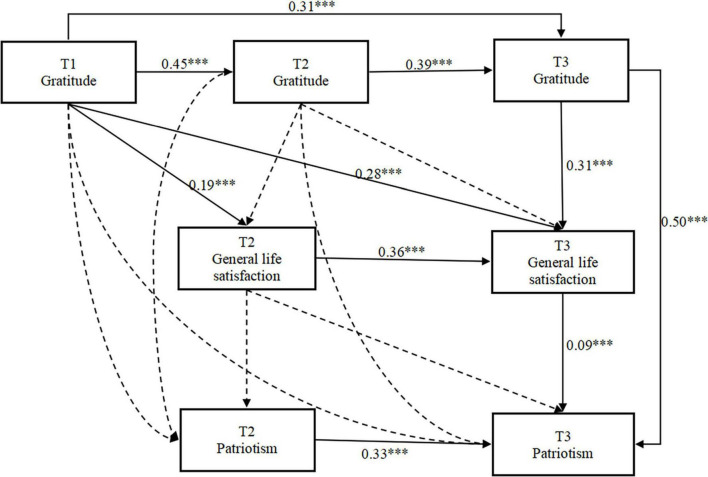
Longitudinal predictive effect of gratitude on patriotism: general life satisfaction as intermediary. The solid line represents statistical significance (*p* < 0.05) and the dashed line represents statistical significance (*p* > 0.05). ***p < 0.001.

Building upon the correlation analysis, we further investigated the temporal lag effects of gratitude, general life satisfaction, and patriotism at three different time points, as depicted in Figure 6. The path coefficients reveal that gratitude levels at Time 1 positively predict gratitude levels at Time 2 (β = 0.45, *p* < 0.001) and Time 3 (β = 0.31, *p* < 0.001), gratitude levels at Time 2 positively predict gratitude levels at Time 3 (β = 0.39, *p* < 0.001). Additionally, general life satisfaction at Time 2 significantly and positively predicts general life satisfaction at Time 3 (β = 0.36, *p* < 0.001), while patriotism at Time 2 similarly predicts patriotism at Time 3 (β = 0.33, *p* < 0.001).

The gratitude at T1 has a complete mediating effect on the patriotism at T3 through the general life satisfaction at T3, while the gratitude at T1 has a partial mediating effect on the patriotism at T3 through the general life satisfaction at T2. Based on the significance of the path coefficient, possible indirect effect paths were tested. The indirect effect analysis adopts the bootstrapping test with deviation correction, reducing the statistical analysis error as much as possible.

Table 5 reveals significant indirect effects for gratitude at T1, mediated through general life satisfaction at T2, on patriotism at T2 (β = 0.10, *P* < 0.001), with a 95% CI of [0.07, 0.13]. Moreover, gratitude at T1 exhibits a significant indirect effect through general life satisfaction at T2 and patriotism at T2, leading to patriotism at T3 (β = 0.04, *P* < 0.001), with a 95% CI of [0.03, 0.05].

**TABLE 5:** The mediating model: indirect effects and 95% confidence intervals.

	Estimate	S.E.	Est/S.E.	95% CI
G-T1 → GLS-T2 → P-T2	0.10***	0.01	6.91	[0.07, 0.13]
G-T1 → GLS-T2 → P-T2→ P-T3	0.04***	0.01	5.77	[0.03, 0.05]

G, gratitude; GLS, general life satisfaction; P, patriotism.

***p < 0.001.

## 3 Discussion

Study 1 in our research was dedicated to exploring the complex relationship between gratitude and patriotism, particularly focusing on how socioeconomic status and general life satisfaction interact within this dynamic. The hypotheses of this study were built around the idea that gratitude positively influences patriotism. Additionally, we hypothesized that general life satisfaction plays a mediating role in the relationship between socioeconomic status and patriotism and that socioeconomic status moderates the link between gratitude and patriotism.

Study 2 took these concepts further by examining the temporal mediation of general life satisfaction in the relationship between gratitude and patriotism. Notably, the results from this study indicated that general life satisfaction acts as a longitudinal mediator between gratitude and patriotism. This finding is significant as it highlights the enduring influence of general life satisfaction in the nexus of gratitude and patriotism over time. The presence of these significant indirect effects emphasizes the pivotal role of general life satisfaction as a mediator in this relationship.

The combination of observed indirect effects and their corresponding confidence intervals contributes to a more nuanced understanding of the relationships among gratitude, general life satisfaction, and patriotism. The findings from this study illuminate the dynamics of how positive emotions like gratitude can impact feelings of national identity, thereby enriching the existing literature in this field of research. This comprehensive approach enhances our understanding of the emotional underpinnings of patriotism and offers valuable insights into the interplay of individual emotions, socioeconomic factors, and patriotism.

### 3.1 The direct effect of gratitude on patriotism

Our study’s findings reveal a statistically significant association between gratitude and patriotism, resonating with the Moral Affect Theory of gratitude (McCullough et al., 2001). This extends the traditional understanding of gratitude’s influence, as previous literature primarily focuses on its impact on interpersonal relationships and individual wellbeing, rather than on patriotism (Hardin, 1993; Michie, 2009; DeSteno et al., 2014, 2019; Dickens and DeSteno, 2016; Rupar et al., 2021). The inclination of grateful individuals toward actions that benefit the collective mirrors a deeper connection with societal values and national identity, suggesting a novel contribution to the field. Although direct research linking gratitude to patriotism is scarce, similar studies have indicated that gratitude can enhance positive collective behaviors (Sasaki et al., 2020), which can be seen as foundational to patriotism. Furthermore, our study highlights gratitude’s role in identity formation among college students, enhancing their sense of belonging and national identity (McCullough et al., 2002). While identity is a component of patriotism (Blank and Schmidt, 2003), the literature has not extensively examined how gratitude specifically influences patriotic identity. Our findings suggest an indirect pathway through which gratitude reinforces patriotism, adding depth to the existing understanding of emotional states and national identity. Despite these contributions, it is important to critically assess this relationship, as the direct link between gratitude and patriotism, while significant, ventures into relatively uncharted territory in the field of emotional and social psychology.

### 3.2 Cross-sectional and longitudinal mediating role of general life satisfaction

Our study demonstrates a significant positive relationship between gratitude and general life satisfaction, both in cross-sectional and longitudinal analyses. This aligns with previous findings that gratitude enhances subjective wellbeing and life satisfaction (Fritz et al., 2019; Armenta et al., 2020). Our data corroborate the notion that gratitude, as a positive experience, contributes to increased life satisfaction (Szczesniak et al., 2019; Zhang, 2020; Lee, 2022). This relationship is further supported by the correlation between gratitude and job satisfaction (Kim et al., 2019; Moon and Jung, 2020), where higher career satisfaction is linked to greater life satisfaction (Hagmaier et al., 2018), and individuals with higher family and personal income report elevated levels of life satisfaction (Wu, 2022).

Moreover, our results indicate that general life satisfaction positively predicts patriotism. This could be attributed to the observation that individuals with high life satisfaction often report strong cultural and ethnic identities (Vietze et al., 2019; Caqueo-Urízar et al., 2021), suggesting a symbiotic relationship between life satisfaction and identity (Dimitrova et al., 2018). Life satisfaction’s significance extends to national legitimacy and political support (Chen and Shi, 2001; Tang, 2016; He et al., 2022), influenced by both institutional political characteristics (Radcliff, 2001; Skitka, 2005; Bjornskov et al., 2007) and individual values and judgments (Diener et al., 1985). Consequently, national attachment, including patriotism and nationalism, is positively correlated with national life satisfaction (Skitka, 2005; Bader, 2006; Liu et al., 2020). Drawing on Social Identity Theory (Tajfel and Turner, 1979), our study suggests that a strong and positive identification with one’s social group, including the nation, is a crucial source of positive self-image. This theory posits that the formation of national/social identity is linked to positive mental health and academic outcomes (Tajfel and Turner, 1986; Cameron, 1999; Smith and Silva, 2011; Rivas-Drake et al., 2014; Reynolds et al., 2017), supporting our hypothesis that general life satisfaction positively predicts patriotism. In summary, our findings contribute to the understanding of the relationship between individual emotional states, general life satisfaction, and national sentiment, offering insights into the complex dynamics of patriotism in the context of emotional and social psychology.

### 3.3 The moderating effect of socioeconomic status

In our cross-sectional samples, we found that socioeconomic status (SES) positively moderated the relationship between gratitude and patriotism. Interestingly, SES itself had a significant negative impact on patriotism, with individuals of lower SES exhibiting more patriotism than those of higher SES. This aligns with previous studies indicating that lower SES is associated with more prosocial behavior and patriotism (Hardin, 1993; Piff et al., 2010; Devos and Sadler, 2019; Tikhonov et al., 2019). This phenomenon might be attributed to the collectivist worldviews often held by lower-status groups, in contrast to the individualistic perspectives prevalent among higher-status groups (Iacoviello and Lorenzi-Cioldi, 2019). Additionally, individuals with lower SES might identify more strongly with national institutions and feel a deeper sense of national identification (Hierro and Rico, 2019).

However, our study also reveals that as SES increases, so does gratitude, which positively impacts patriotism. This suggests that gratitude’s influence on patriotism is more pronounced among individuals with higher SES. According to Fredrickson’s broaden-and-build theory (Fredrickson, 2001), gratitude expands an individual’s thought-action repertoire and builds various personal resources, including social support systems, which can enhance prosocial behavior and social adaptability. The enhancement of gratitude, particularly among college students, is linked to the development of positive values and a stronger sense of belonging and identity (McCullough et al., 2002). Consequently, the negative impact of SES on patriotism diminishes with increased levels of gratitude, leading to a quicker rise in patriotism among those with higher SES.

Contrary to our expectations, the hypothesis that SES moderates the relationship between gratitude and patriotism in the second moderation path was not supported. The results indicated that this moderation path was not significant, suggesting that the increase in patriotism as life satisfaction grows is similar across different SES groups. This finding is somewhat at odds with previous research suggesting that life satisfaction has a greater positive impact on patriotism among lower SES individuals (Vayness et al., 2020). Studies have also shown a positive correlation between individual life satisfaction and national attachment (Skitka, 2005; Liu et al., 2020), reflecting an innate human need for attachment and recognition (Bader, 2006). Our findings suggest that while an increase in life satisfaction boosts patriotism, the effect does not significantly differ between high and low-SES groups. Furthermore, the lack of significant moderating effects in the longitudinal samples implies that the relationships between gratitude, life satisfaction, and patriotism remained stable over time, regardless of SES levels. This could be due to limited variation in the moderating factors or the presence of unaccounted confounding variables. The stability of these relationships over time highlights the enduring nature of the connections between these variables, underscoring the complex interplay of emotional states, general life satisfaction, and patriotism.

### 3.4 Strengths and limitations

Our study makes a significant contribution to the field of patriotism research by examining the roles of general life satisfaction and gratitude within the context of social class. This approach offers a fresh perspective, highlighting how socioeconomic factors intertwine with emotional and psychological aspects to influence patriotism. Notably, our findings reveal that high levels of gratitude and general life satisfaction correlate with increased patriotism. This is particularly pronounced among individuals with low socioeconomic status, where improvements in general life satisfaction have a substantial impact on patriotism. Conversely, for those with higher socioeconomic status, an increase in gratitude levels appears to be more influential in enhancing patriotism. These insights provide valuable guidance for policymakers and educators in fostering patriotism through targeted strategies that address the specific needs and characteristics of different socioeconomic groups. These findings have practical implications, suggesting that tailored interventions focusing on enhancing general life satisfaction and gratitude could effectively foster patriotism in targeted populations.

Our study, however, is not without limitations. The reliance on perceived subjective social class, without the inclusion of objective measures, may not fully capture the nuanced relationship between social class and patriotism. Future research could benefit from incorporating both subjective and objective assessments of social class to provide a more comprehensive understanding. Additionally, the sensitive nature of patriotism as a research topic suggests that our reliance on subjective reporting may not fully reflect true attitudes. Incorporating methods to assess implicit attitudes could offer a more rounded view of patriotism. Furthermore, the generalizability of our findings is limited by the predominance of college students in our sample, indicating the need for more diverse and representative sampling in future studies.

Future research should explore the dynamics of patriotism across a broader demographic spectrum, including various age groups, professions, and cultural backgrounds. Investigating the long-term effects of gratitude and general life satisfaction on patriotism, and how these relationships evolve, would also be valuable. Our study underscores the importance of considering both emotional states and socioeconomic factors in understanding and fostering patriotism. The key takeaway is that patriotism is a multifaceted sentiment influenced by a complex interplay of individual emotions, general life satisfaction, and social class. Recognizing and addressing these diverse influences can aid in developing more effective strategies to nurture a sense of national pride and unity. In summary, while our study offers valuable insights into the factors influencing patriotism, it is important to consider these limitations when interpreting our findings. Acknowledging and addressing these limitations can pave the way for more comprehensive and robust research in the future.

## Data availability statement

The raw data supporting the conclusions of this article will be made available by the authors, without undue reservation.

## Ethics statement

The studies involving humans were approved by the Institutional Review Board (or Ethics Committee) of Wenzhou University of Technology. The studies were conducted in accordance with the local legislation and institutional requirements. Written informed consent to participate in this study was not required from the participants in accordance with the national legislation and the institutional requirements.

## Author contributions

YH and GC performed material preparation, data collection, and analysis. YH and HZ wrote the first draft of the manuscript. GC provided advice on writing the final draft. HZ, QL, and WZ completed the final draft. All authors contributed to the study’s conception and design, commented on previous versions of the manuscript, and read and approved the final manuscript.

## References

[B1] Adler N. E. Epel E. S. Castellazzo G. Ickovics J. R. (2000). Relationship of subjective and objective social status with psychological and physiological functioning: preliminary data in healthy, white women. *Health Psychol.* 19 586–592. 10.1037//0278-6133.19.6.586 11129362

[B2] Ariely G. (2021). Living the past? do historical legacies moderate the relationship between national chauvinism/cultural patriotism and xenophobic attitudes toward immigrants. *Int. J. Comp. Sociol.* 1–23.

[B3] Armenta C. N. Fritz M. M. Walsh L. C. Lyubomirsky S. (2020). Satisfied yet striving: gratitude promotes life satisfaction and improvement motivation in youth. *Emotion* 22 1004–1016. 10.1037/emo0000896 32915004

[B4] Bader M. J. (2006). The psychology of patriotism. *Phi Delta Kappan.* 87 582–584.

[B5] Bartlett M. Y. Condon P. Cruz J. Baumann J. DeSteno D. (2012). Gratitude: prompting behaviours that build relationships. *Cogn. Emot.* 26 2–13. 10.1080/02699931.2011.561297 21500044

[B6] Bartlett M. Y. DeSteno D. (2006). Gratitude and prosocial behavior: helping when it costs you. *Psychol. Sci.* 17 319–325. 10.1111/j.1467-9280.2006.01705.x 16623689

[B7] Bas M. (2016). The evaluation of the university students’ patriotism levels according to gender, age, family structure, and sports activities. *Eur. J. Educ. Stud.* 2 34–43.

[B8] Bjornskov C. Dreher A. Fischer J. A. V. (2007). The bigger the better? evidence of the effect of government size on life satisfaction around the world. *Public Choice* 127 267–292.

[B9] Blanchflower D. G. Oswald A. J. (2000). Well-being over time in Britain and the USA. *J. Public Econ.* 88 1359–1386.

[B10] Blank T. Schmidt P. (2003). National identity in a united Germany: Nationalism or patriotism? an empirical test with representative data. *Polit. Psychol.* 24 289–312.

[B11] Brown M. Chua K. J. Lukaszewski A. W. (2021). Formidability and socioeconomic status uniquely predict militancy and political moral foundations. *Pers. Individ. Dif.* 168:110284.

[B12] Buschor C. Proyer R. T. Ruch W. (2013). Self- and peer-rated character strengths: how do they relate to satisfaction with life and orientations to happiness? *J. Posit. Psychol.* 8 116–127.

[B13] Cameron J. E. (1999). Social identity and the pursuit of possible selves: implications for the psychological well-being of university students. *Group Dyn. Theory Res. Pract.* 3 179–189.

[B14] Caqueo-Urízar A. Flores J. Mena-Chamorro P. Urzúa A. Irarrázaval M. (2021). Ethnic identity and life satisfaction in indigenous adolescents: the mediating role of resilience. *Child. Youth Serv. Rev.* 120:105812.

[B15] Carvalho C. L. Pinto I. R. Marques J. M. (2021). The nationalist movements in Spain, today: a catalonian and Basque comparison. *Rev. Psicol.* 39 687–715.

[B16] Chen X. Y. Shi T. J. (2001). Media effects on political confidence and trust in the People’s Republic of China in the post-Tiananmen period. *East Asia* 19 84–118.

[B17] Chen Y. H. Bai L. Li L. F. (2015). The characteristics and development of moral emotion and its influence on moral behavior. *Stud. Psychol. Behav.* 13 627–636.

[B18] DeSteno D. Duong F. Lim D. Kates S. (2019). The grateful don’t cheat Gratitude as a fount of virtue. *Psychol. Sci.* 30 979–988. 10.1177/0956797619848351 31145653

[B19] DeSteno D. Li Y. Dickens L. Lerner J. S. (2014). Gratitude: a tool for reducing economic impatience. *Psychol. Sci.* 25 1262–1267. 10.1177/0956797614529979 24760144

[B20] Devos T. Sadler M. (2019). Context diversity predicts the extent to which the American identity is implicitly associated with Asian Americans and European Americans. *Asian Am. J. Psychol.* 10 182–193.

[B21] Dickens L. DeSteno D. (2016). The grateful are patient: heightened daily gratitude is associated with attenuated temporal discounting. *Emotion* 16 421–425. 10.1037/emo0000176 27018609

[B22] Diener E. Emmons R. A. Larsen R. J. Griffin S. (1985). The satisfaction with life scale. *J. Pers. Assess.* 49 71–75. 16367493 10.1207/s15327752jpa4901_13

[B23] Dimitrova R. Buzea C. Tauová J. U. F. Zakaj S. Crocetti E. (2018). Relationships between identity domains and life satisfaction in minority and majority youth in Albania, Bulgaria, Czech Republic, Kosovo, and Romania. *Eur. J. Dev. Psychol.* 15 61–82.

[B24] Draž,anová L. Roberts A. (2023). National attachments and good citizenship: a double-edged sword. *Polit. Stud.* 1–24..

[B25] Easterlin R. A. (1995). Will raising the incomes of all increase the happiness of all? *J. Econ. Behav. Organ.* 27 35–47.

[B26] Emerson S. D. Guhn M. Gadermann A. M. (2017). Measurement invariance of the satisfaction with life scale: reviewing three decades of research. *Qual. Life Res.* 26 2251–2264. 28324322 10.1007/s11136-017-1552-2

[B27] Emmons R. A. McCullough M. E. (2004). *The Psychology of Gratitude.* New York, NY: Oxford University Press.

[B28] Emmons R. A. McCullough M. E. Tsang J. A. (2003). “The measurement of gratitude,” in *Handbook of Positive Psychology Assessment*, eds Lopez S. Snyder C. R. (Washington, DC: American Psychological Association).

[B29] Fagley N. S. Adler M. G. (2012). Appreciation: a spiritual path to finding value and meaning in the workplace. *J. Manag. Spirituality Relig.* 9 167–187.

[B30] Flavin P. Keane M. J. (2012). Life satisfaction and political participation: evidence from the United States. *J. Happiness Stud.* 13 63–78.

[B31] Fredrickson B. L. (2001). The role of positive emotions in positive psychology: the broaden-and-build theory of positive emotions. *Am. Psychol.* 56 218–226. 11315248 10.1037//0003-066x.56.3.218 PMC3122271

[B32] Fritz M. M. Armenta C. N. Walsh L. C. Lyubomirsky S. (2019). Gratitude facilitates healthy eating behavior in adolescents and young adults. *J. Exp. Soc. Psychol.* 81 4–14.

[B33] Gomez Berrocal M. D. C. Porras C. Mata S. (2020). The role of group identification in the well-being of Spaniards with gypsy ethnicity. *J. Soc. Psychol.* 160 204–215. 10.1080/00224545.2019.1634504 31258025

[B34] Goodman E. Maxwell S. Malspeis S. Adler N. (2015). Developmental trajectories of subjective social status. *Pediatrics* 136 633–640. 10.1542/peds.2015-1300 PMC4552092 26324868

[B35] Gordon A. M. Chen S. (2013). Does power help or hurt? the moderating role of self-other focus on power and perspective-taking in romantic relationships. *Pers. Soc. Psychol. Bull.* 39 1097–1110. 23748962 10.1177/0146167213490031

[B36] Grant A. M. Gino F. (2010). A little thanks goes a long way: explaining why gratitude expressions motivate prosocial behavior. *J. Pers. Soc. Psychol.* 98 946–955. 10.1037/a0017935 20515249

[B37] Hagmaier T. Abele A. E. Goebel K. (2018). How do career satisfaction and life satisfaction associate? *J. Manag. Psychol.* 33 142–160.

[B38] Hamada T. Shimizu M. Ebihara T. (2021). Good patriotism, social consideration, environmental problem cognition, and pro-environmental attitudes and behaviors: a cross-sectional study of Chinese attitudes. *SN Appl. Sci.* 3:361.

[B39] Hardin R. (1993). Altruism and mutual advantage. *Soc. Serv. Rev.* 67 358–373.

[B40] He L. Wang K. Liu T. Li T. Zhu B. (2022). Does political participation help improve the life satisfaction of urban residents: empirical evidence from China. *PLoS One* 17:e0273525. 10.1371/journal.pone.0273525 36201453 PMC9536587

[B41] Hierro M. J. Rico G. (2019). Economic crisis and national attitudes: experimental evidence from Spain. *Ethn. Racial Stud.* 42 820–837.

[B42] Huang S. H. Guan Y. X. Zhang W. (2014). Development of a moral emotion questionnaire for college students. *Psychol. Behav. Stud.* 12 521–526.

[B43] Huang Z. Yang Z. Meng T. (2023). National identity of locality: the state, patriotism, and nationalism in Cyber China. *J. Chin. Polit. Sci.* 28 51–83.

[B44] Iacoviello V. Lorenzi-Cioldi F. (2019). Collectivism and individualism in status hierarchies: socialization and social identity explanations. *Int. Rev. Soc. Psychol.* 32 2–4.

[B45] Ishio Y. (2010). Social bases of American patriotism: examining effects of dominant social statuses and socialization. *Curr. Sociol.* 58 67–93.

[B46] Jackman M. R. Jackman R. W. (1973). An interpretation of the relation between objective and subjective social status. *Am. Sociol. Rev.* 38 569–582. 4745630

[B47] Karakaya Y. (2022). “The Panorama of Conquest”: a cultural approach to national emotions. *Sociol. Forum* 37 789–811.

[B48] Kim S. R. Park O. L. Kim H. Y. Kim J. Y. (2019). Factors influencing well-being in clinical nurses: a path analysis using a multi-mediation model. *J. Clin. Nurs.* 28 4549–4559. 10.1111/jocn.15045 31468601

[B49] Kraus M. W. Piff P. K. Keltner D. (2011). Socioeconomic status as culture: the convergence of resources and rank in the social realm. *Curr. Dir. Psychol. Sci.* 20 246–250.

[B50] Lambert N. M. Graham S. M. Fincham F. D. (2009). A prototype analysis of gratitude: varieties of gratitude experiences. *Pers. Soc. Psychol. Bull.* 35 1193–1207. 10.1177/0146167209338071 19581434

[B51] Lau B. H. P. Cheng C. (2017). Gratitude and coping among familial caregivers of persons with dementia. *Aging Ment. Health* 21 445–453. 10.1080/13607863.2015.1114588 26613417

[B52] Lavelock C. R. Griffin B. J. Worthington E. L. Benotsch E. G. Lin Y. Greer C. L. (2016). A qualitative review and integrative model of gratitude and physical health. *J. Psychol. Theol.* 44 55–86. 10.3389/fpubh.2023.1092145 36950093 PMC10025337

[B53] Lee B. (2022). A serial mediation model of gratitude on life satisfaction in people with multiple sclerosis: the intermediary role of perceived stress and mental health symptoms. *Mult. Scler. Relat. Disord.* 58:103421. 10.1016/j.msard.2021.103421 35216777

[B54] Leung J. P. Leung K. (1992). Life satisfaction, self-concept, and relationship with parents in adolescence. *J. Youth Adolesc.* 21 653–665. 24264168 10.1007/BF01538737

[B55] Li B. Hu X. Chen L. Wu C. (2023). Longitudinal relations between school climate and prosocial behavior: the mediating role of gratitude. *Psychol. Res. Behav. Manag.* 16 419–430. 36819008 10.2147/PRBM.S395162 PMC9936877

[B56] Liu X. Zhang Y. Vedlitz A. (2021). Political values and life satisfaction in China. *China Q.* 245 276–291.

[B57] Liu X. S. Zhang Y. L. Vedlitz A. (2020). Political values and life satisfaction in China. *China Q.* 245 276–291.

[B58] Mackie D. M. Devos T. Smith E. R. (2000). Intergroup emotions: explaining offensive action tendencies in an intergroup context. *J. Pers. Soc. Psychol.* 79 602–616. 11045741

[B59] Martynov M. Y. Fadeeva L. A. Gaberkorn A. I. (2020). Patriotism as political discourse in contemporary Russia. *Polis Polit. Stud.* 2 109–121.

[B60] McCullough M. E. Emmons R. A. Tsang J. A. (2002). The grateful disposition: a conceptual and empirical topography. *J. Pers. Soc. Psychol.* 82 112–127. 11811629 10.1037//0022-3514.82.1.112

[B61] McCullough M. E. Kilpatrick S. D. Emmons R. A. Larson D. B. (2001). Is gratitude a moral affect? *Psychol. Bull.* 127 249–266. 10.1037/0033-2909.127.2.249 11316013

[B62] Meltzer A. H. Richard S. F. (1981). A rational theory of the size of government. *J. Polit. Econ.* 89 914–927.

[B63] Michie S. (2009). Pride gratitude: how positive emotions influence the prosocial behaviors of organizational leaders. *J. Leadersh. Organ. Stud.* 15 393–403.

[B64] Moon H. Jung M. (2020). The relationship between a disposition of gratitude, clinical stress, and clinical satisfaction in nursing students. *Perspect. Psychiatr. Care* 56 768–776. 10.1111/ppc.12491 32109327

[B65] Mummendey A. Klink A. Brown R. (2001). Nationalism and patriotism: national identification and out-group rejection. *Br. J. Soc. Psychol.* 40 159–172. 10.1348/014466601164740 11446222

[B66] Osborne D. Milojev P. Sibley C. G. (2017). Authoritarianism and national identity: examining the longitudinal effects of SDO and RWA on nationalism and patriotism. *Pers. Soc. Psychol. Bull.* 43 1086–1099. 10.1177/0146167217704196 28903711

[B67] Pang Y. Song C. Ma C. (2022). Effect of different types of empathy on prosocial behavior: gratitude as mediator. *Front. Psychol.* 13:768827. 10.3389/fpsyg.2022.768827 35250712 PMC8891453

[B68] Pankaew A. Thananithichot S. Satidporn W. (2022). Determinants of political participation in Thailand: an analysis of survey data (2002–2014). *Asian Politics Policy* 14 92–113.

[B69] Peña Y. Sidanius J. (2002). US patriotism and ideologies of group dominance: a tale of asymmetry. *J. Soc. Psychol.* 142 782–790. 10.1080/00224540209603936 12450351

[B70] Perez J. A. Peralta C. O. Besa F. B. (2021). Gratitude and life satisfaction: the mediating role of spirituality among Filipinos. *J. Beliefs Values* 42 511–522.

[B71] Piff P. K. Kraus M. W. Côté S. Cheng B. H. Keltner D. (2010). Having less, giving more: the influence of social class on prosocial behavior. *J. Pers. Soc. Psychol.* 99 771–784. 20649364 10.1037/a0020092

[B72] Radcliff B. (2001). Politics, markets, and life satisfaction: the political economy of human happiness. *Am. Polit. Sci. Rev.* 95 939–962. 10.3390/ijerph15050900 29751489 PMC5981939

[B73] Reynolds K. J. Lee E. Turner I. Bromhead D. Subasic E. (2017). How does school climate impact academic achievement? an examination of social identity processes. *Sch. Psychol. Int.* 38 78–97.

[B74] Rivas-Drake D. Seaton E. K. Markstrom C. Quintana S. Syed M. Lee R. M. (2014). Ethnic and racial identity in adolescence: implications for psychosocial, academic, and health outcomes. *Child Dev.* 85 40–57. 24490891 10.1111/cdev.12200 PMC6673646

[B75] Rubin M. Stuart R. (2018). Kill or cure? different types of social class identification amplify and buffer the relation between social class and mental health. *J. Soc. Psychol.* 158 236–251. 28481719 10.1080/00224545.2017.1327405

[B76] Rupar M. Jamróz-Dolińska K. Kołeczek M. Sekerdej M. (2021). Is patriotism helpful to fight the crisis? the role of constructive patriotism, conventional patriotism, and glorification amid the COVID-19 pandemic. *Eur. J. Soc. Psychol.* 51 862–877. 10.1002/ejsp.2777 34219823 PMC8239923

[B77] Sasaki E. Jia L. Lwa H. Y. Goh M. T. (2020). Gratitude inhibits competitive behaviour in threatening interactions. *Cogn. Emot.* 34 1097–1111. 10.1080/02699931.2020.1724892 32026747

[B78] Schoon I. Cheng H. Gale C. R. Batty G. D. Deary I. J. (2010). Social status, cognitive ability, and educational attainment as predictors of liberal social attitudes and political trust. *Intelligence* 38 144–150.

[B79] Sharma K. Hooda M. (2023). The new elective of national cadet corps (NCC): a review of literature. *Int. J. Res. Innov. Soc. Sci.* 7 384–396.

[B80] Singh-Manoux A. Marmot M. G. Adler N. E. (2005). Does subjective social status predict health and change in health status better than objective status? *Psychosom. Med.* 67 855–861. 16314589 10.1097/01.psy.0000188434.52941.a0

[B81] Skitka L. J. (2005). Patriotism or nationalism? understanding post-September 11, 2001, flag display behavior. *J. Appl. Soc. Psychol.* 35 1995–2011.

[B82] Smith T. B. Silva L. (2011). Ethnic identity and personal well-being of people of color: a Meta analysis. *J. Couns. Psychol.* 58 42–60. 21171745 10.1037/a0021528

[B83] Smith T. W. Kim S. (2006). National pride in comparative perspective 1995/96 and 2003/04. *Int. J. Public Opin. Res.* 18 127–136.

[B84] Spinner-Halev J. Theiss-Morse E. (2003). National identity and self-esteem. *Perspect. Polit.* 1 515–632.

[B85] Stieger M. Hill P. L. Allemand M. (2019). Looking on the bright side of life: gratitude and experiences of interpersonal transgressions in adulthood and daily life. *J. Pers.* 88 430–446. 10.1111/jopy.12501 31309550

[B86] Stutzer A. Bruno S. F. (2006). Political participation and procedural utility: an empirical study. *Eur. J. Polit. Res.* 45 391–418.

[B87] Sun P. Jiang H. Chu M. Qian F. (2014). Gratitude and school well-being among Chinese university students: interpersonal relationships and social support as mediators. *Soc. Behav. Pers.* 42 1689–1698.

[B88] Szczesniak M. G. Bielecka I. Bajkowska A. Czaprowska D. M. (2019). Religious/spiritual struggles and life satisfaction among young Roman Catholics: the mediating role of gratitude. *Religions* 10:395.

[B89] Tajfel H. Turner J. C. (1979). “An integrative theory of intergroup conflict,” in *Social Psychology of Intergroup Relations*, eds Austin W. G. Worchel S. (Monterey, CA: Brooks).

[B90] Tajfel H. Turner J. C. (1986). “The social identity theory of intergroup behavior,” in *Psychology of Intergroup Relation*, eds Worchel S. Austin W. G. (Chicago, IL: Hall Publishers).

[B91] Tang W. F. (2016). *Populist Authoritarianism: Chinese Political Culture and Regime Sustainability.* New York, NY: Oxford university press.

[B92] Tikhonov A. A. Espinosa A. Huynh Q. L. Anglin D. M. (2019). Bicultural identity harmony and American identity are associated with positive mental health in US racial and ethnic minority immigrants. *Cult. Divers. Ethn. Minor. Psychol.* 25 494–504. 10.1037/cdp0000268 30816754

[B93] Vayness J. Duong F. DeSteno D. (2020). Gratitude increases third party punishment. *Cogn. Emot.* 35 1020–1027. 10.1080/02699931.2019.1700100 31814516

[B94] Vietze J. Juang L. P. Schachner M. K. (2019). Peer cultural socialisation: a resource for minority students’ cultural identity, life satisfaction, and school values. *Intercult. Educ.* 30 579–598.

[B95] Ward G. (2020). Happiness and voting: evidence from four decades of elections in Europe. *Am. J. Polit. Sci.* 64 504–518.

[B96] Ward G. De Neve J. E. Ungar L. H. Eichstaedt J. C. (2021). (Un)happiness and voting in U.S. presidential elections. *J. Pers. Soc. Psychol.* 120 370–383. 10.1037/pspi0000249 32700960

[B97] Wei C. Li Q. Lian Z. Luo Y. Song S. Chen H. (2022). Variation in public trust, perceived societal fairness, and well-being before and after COVID-19 onset—Evidence from the China family panel studies. *Int. J. Environ. Res. Public Health.* 19:12365. 10.3390/ijerph191912365 36231662 PMC9566506

[B98] Weitz-Shapiro R. Matthew W. (2011). The link between voting and life satisfaction in Latin America. *Lat. Am. Polit. Soc.* 53 101–126.

[B99] Williams R. L. Foster L. N. Krohn K. R. (2008). Relationship of patriotism measures to critical thinking and emphasis on civil liberties versus national security. *Anal. Soc. Issues Public Policy* 8 139–156.

[B100] Wodak R. (2009). *Discursive Construction of National Identity.* Edinburgh: Edinburgh University Press.

[B101] Wood A. M. Froh J. J. Geraghty A. W. (2010). Gratitude and well-being: a review and theoretical integration. *Clin. Psychol. Rev.* 30 890–905. 20451313 10.1016/j.cpr.2010.03.005

[B102] Wu Q. (2022). Individual versus household income and life satisfaction: the moderating effects of gender and education. *J. Comp. Fam. Stud.* 52 668–688.

[B103] Yost-Dubrow R. Dunham Y. (2018). Evidence for a relationship between trait gratitude and prosocial behaviour. *Cogn. Emot.* 32 397–403. 10.1080/02699931.2017.1289153 28278738

[B104] Zhang J. W. (2020). Grateful people are happier because they have fond memories of their past. *Pers. Individ. Differ.* 152:109602.

[B105] Zhang L. Li W. Ye Y. Yang K. Jia N. Kong F. (2022). Being grateful every day will pay off: a daily diary investigation on relationships between gratitude and well-being in Chinese young adults. *J. Posit. Psychol.* 18 853–865.

[B106] Zhang Y. R. Zuo B. (2012). The relationship between Chinese nation identity and national pride, in-group favoritism of adolescents. *China J. Health Psychol.* 20 86–88.

[B107] Zhang Z. Q. Zhu R. D. Liu C. (2020). What are the effects of national pride on prosocial behaviors? the moderating influences of group type and loyalty (in Chinese). *Chin. Sci. Bull.* 65 1956–1966.

